# Should I Stay or Should I Go? Cognitive Modeling of Left-Turn Gap Acceptance Decisions in Human Drivers

**DOI:** 10.1177/00187208221144561

**Published:** 2022-12-19

**Authors:** Arkady Zgonnikov, David Abbink, Gustav Markkula

**Affiliations:** 2860 Delft University of Technology, Netherlands; 4468 University of Leeds, UK

**Keywords:** driver behavior, decision making, computational modeling

## Abstract

**Objective:**

We aim to bridge the gap between naturalistic studies of driver behavior and modern cognitive and neuroscientific accounts of decision making by modeling the cognitive processes underlying left-turn gap acceptance by human drivers.

**Background:**

Understanding decisions of human drivers is essential for the development of safe and efficient transportation systems. Current models of decision making in drivers provide little insight into the underlying cognitive processes. On the other hand, laboratory studies of abstract, highly controlled tasks point towards noisy evidence accumulation as a key mechanism governing decision making. However, it is unclear whether the cognitive processes implicated in these tasks are as paramount to decisions that are ingrained in more complex behaviors, such as driving.

**Results:**

The drivers’ probability of accepting the available gap increased with the size of the gap; importantly, response time increased with time gap but not distance gap. The generalized drift-diffusion model explained the observed decision outcomes and response time distributions, as well as substantial individual differences in those. Through cross-validation, we demonstrate that the model not only explains the data, but also generalizes to out-of-sample conditions.

**Conclusion:**

Our results suggest that dynamic evidence accumulation is an essential mechanism underlying left-turn gap acceptance decisions in human drivers, and exemplify how simple cognitive process models can help to understand human behavior in complex real-world tasks.

**Application:**

Potential applications of our results include real-time prediction of human behavior by automated vehicles and simulating realistic human-like behaviors in virtual environments for automated vehicles.

## INTRODUCTION

Understanding the seemingly mundane decisions made daily by millions of human drivers is critical for designing traffic infrastructure and engineering automated vehicles which can safely interact with humans around them. Overtaking a slow-moving vehicle, merging onto a highway, taking a left turn across path of an oncoming car—all these situations require the driver to make an informed and well-timed decision whether or not to accept a space- and time-gap. Gap acceptance models have been instrumental in clarifying which factors affect these decisions. These models, developed with support from both naturalistic data and driving simulator studies, typically describe what information drivers use, and predict *what* decisions they make (Davis & Swenson, 2004; Farah et al., 2009; Hamed et al., 1997; Toledo, 2007). Still, little is known about *how* the drivers process the relevant perceptual information while arriving to a decision. Understanding and modeling the underlying cognitive mechanisms can lead to more generalizable predictions of these decisions and help to predict how dynamic changes in the environment over the time course of a decision affect the behavior (Jarecki et al.,2020).

Laboratory studies of abstract, highly controlled tasks have pointed towards noisy evidence accumulation as the primary mechanism governing human decision making (Gold & Shadlen, 2007; Ratcliff & McKoon, 2008; Stine et al., 2020). However, it is unclear whether the mechanisms implicated in simple perceptual decisions in the laboratory are as paramount to decisions that are part of complex behaviors like driving. In tasks such as gap acceptance, the available perceptual information is much richer, often varying continuously over time, and motor behaviors are more complex than in traditional laboratory-based decision-making tasks such as motion or orientation discrimination. Even carefully designed abstract tasks can deprive human subjects of the potentially relevant context, the senses of agency and embodiment (Matusz et al., 2019; Shamay-Tsoory & Mendelsohn, 2019). This could hinder understanding of the cognitive mechanisms of interest, and it is far from obvious how to generalize from simple laboratory paradigms on perceptual choice to driving situations. Recently, first steps have been taken toward applying evidence accumulation models in driving and road traffic contexts, but these have been limited to speeded responses to discrete stimuli (Ratcliff, 2015; Ratcliff & Strayer, 2014), or have not been fully stringent in model analyses (Giles et al., 2019; Markkula, Boer, et al., 2018; Xue et al., 2018). Therefore, there is currently a lack of principled investigations of evidence accumulation in contexts where drivers need to consider more complex traffic situations where the correct response is not immediately clear from the stimulus.

The aim of this paper is to bridge the gap between studies of human road user behavior and models of decision making elaborated in basic cognitive science. To this end, we developed an experimental method that allowed drivers to make a decision that is very common in everyday driving—accepting or rejecting a gap when turning across oncoming traffic—but in a way that simultaneously permitted stringent modeling. Using this method, we measured how the participants’ decisions and response times varied with distance and time-to-arrival of the oncoming vehicle. We developed a model to capture the underlying cognitive processes determining the gap acceptance decisions, representing the drivers’ decision making as a dynamic process based on accumulation of noisy evidence over time. We then fitted the developed model to the individual participants’ data. The model captured the between- and within-participant variability in decision outcomes and response times, and successfully predicted the observed behavior in out-of-sample conditions. The results suggest that evidence accumulation of distance and time-to-arrival under time pressure underlies drivers’ decision making during left-turn gap acceptance, and illustrate how simple cognitive models can explain human road user behavior.

## METHOD

This research complied with the tenets of the Declaration of Helsinki and was approved by the Human Research Ethics Committee of TU Delft. Informed consent was obtained from each participant.

### Participants

Sixteen participants (mean age 27 (range: 22 to 34) years old; 10 male, 6 female) performed a virtual driving task in exchange for a gift voucher worth €15. The participants had been in possession of a driving license for 8 (range: 3 to 18) years on average, of which they have been driving regularly (as interpreted by the participant) for the average of 5 (range: 0 to 18) years.

### Setup

**Figure 1.**
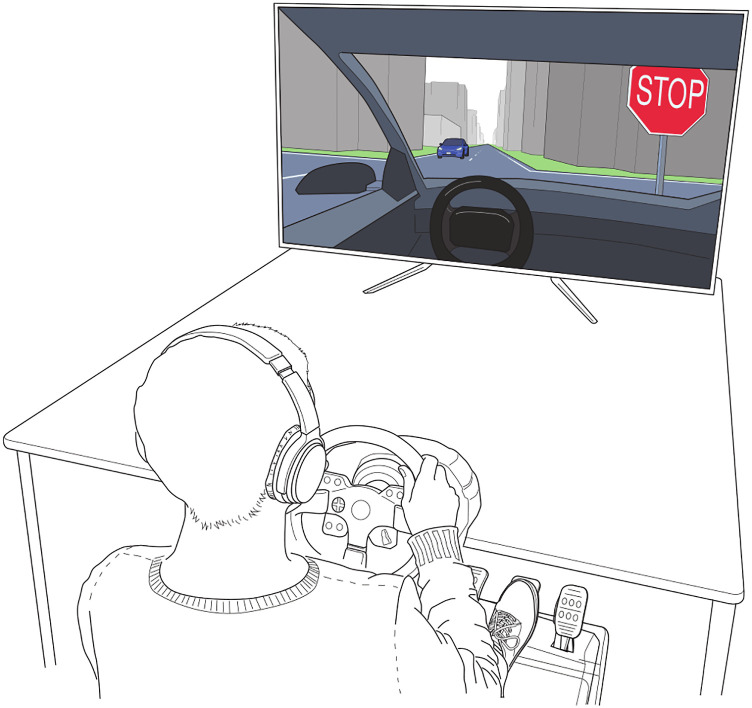
Experimental setup.

The participants performed the experiment in a first-person-view fixed-base driving simulator, which included a 65-inch screen and a commercially available Logitech G29 steering wheel (Figure 1). The distance between the center of the screen and the participants’ eyes was approximately 1.5 m. Carla (Dosovitskiy et al., 2017) was used as a simulation software.

### Protocol

Each participant was asked to drive in a virtual urban area (1.5 by 1.5 km, a regular grid of square blocks 150 by 150 m each), following eight randomly generated routes. Each route included 25 intersections; the participants were instructed to go straight on 5 intersections, turn right on 5 intersections, and turn left on 15 intersections (the order of the turns was randomized). This resulted in 120 left turns per participant in total.

The instructions were provided to the participants via auditory navigation prompts which were repeated twice, 120 m and 30 m before the intersection. The participants were told that there will be traffic coming from the opposite direction, and were instructed to fully stop the vehicle at the stopping line on each intersection, which was also marked by the stop sign.

**Figure 2.**
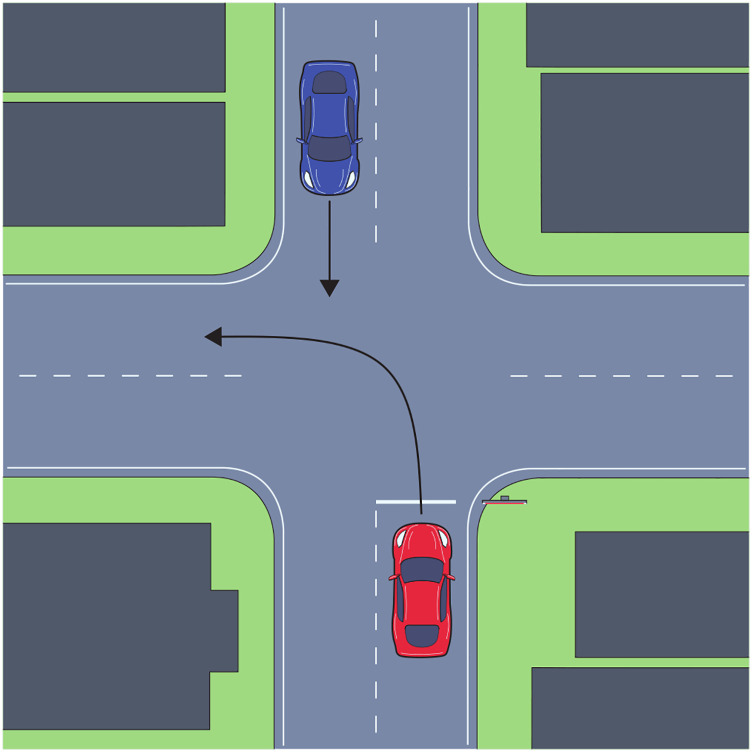
Top-down view of the left-turn interaction.

During left turn trials (Figure 2), at the moment the driver stopped, an oncoming vehicle instantaneously appeared across the intersection at a distance of 90, 120, or 150 m (randomly chosen for each trial independently) from the ego vehicle. The initial time-to-arrival (TTA, 4, 5, or 6 s) of the oncoming vehicle was randomly chosen for each left turn. Time-to-arrival conditions were balanced within each route, such that on each route there were exactly five left turns at each of the three time-to-arrival levels. To summarize, we used a 3-by-3 factorial design, varying initial time-to-arrival and distance to the oncoming vehicle.

The speed of the oncoming vehicle at the moment of its appearance at the intersection was calculated as the ratio of initial distance to initial time-to-arrival (Table 1), and was held constant at that level throughout the trial. The participants were required to decide whether to *go* before the oncoming car arrived, or *stay*, that is, wait until the car passed the intersection and only then continue the route. Positions, velocities, and accelerations of the two vehicles during the interaction were recorded at 100 Hz.

**TABLE 1:** Experimental Conditions and the Associated Values of the Speed (m/s) of the Oncoming Vehicle.

distance∖TTA	4s	5s	6s
90m	22.5	18	15
120m	30	24	20
150m	37.5	30	25

### Data Analysis

**Figure 3.**
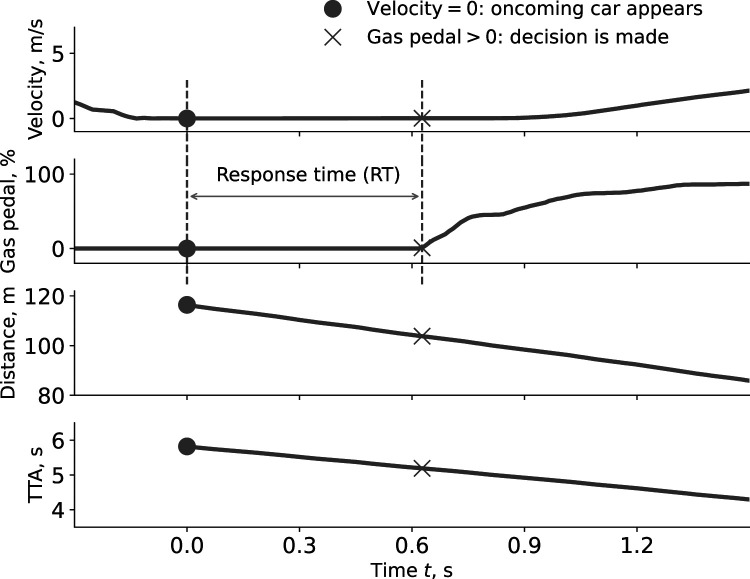
Time traces of a typical interaction, showing the participant decelerating for the stop sign, the oncoming car appearing at a constant speed at *t* = 0, the dynamics of distance and time-to-arrival (TTA), and the moment when the participant presses the gas pedal to cross in front of the oncoming car.

Initial time-to-arrival and distance were the independent variables. Decision (go or stay) and response time (only for “go” decisions) were the dependent variables. The decision was determined based on whether the participant crossed the intersection before the oncoming vehicle. Response time was defined as the time between the appearance of the oncoming vehicle and the time when the participant first pressed the gas pedal after the oncoming vehicle appeared (Figure 3). For “turn” decisions, response time therefore combined the time it took for the driver to make a decision and perceptual and motor delays directly associated with the decision. For “stay” decisions however, appropriate response time measure could not be extracted from the data because unlike “go” decisions, there are no cues in the recorded data that mark the moment when the “stay” decision was made.

In this paper, we focus on left-turn gap acceptance decisions. We therefore did not analyze the trials in which the participants did not evaluate a gap, which was the case if they a) performed right turns; b) went straight through the intersection, or c) did not fully stop before the left turn so the oncoming car did not appear at the intersection; the latter happened in 27 (1.4%) left turns. Finally, we excluded 10 (0.5%) “go” decisions in which response time could not be determined because the participant had the gas pedal pressed throughout the trial, and 1 left-turn decision with extreme response time (greater than 2s). This resulted in 1878 analyzed left turns.

### Statistical Analysis

Mixed-effects models were used to analyze the effects of distance and TTA on decision outcome (binomial model) and response time (linear model); the models were implemented using R package lme4. In both models, random effects of participant were included to account for individual differences, with the maximum random effects structure permitting model convergence (see the analysis scripts for details). “Stay” decisions were coded as 0, “go” decisions as 1.

### Data and Code Availability

Supplementary information, data collection code, analysis scripts, and the data are available at https://osf.io/x3ns6/.

## EXPERIMENTAL RESULTS

On average, the participants decided to turn before the arrival of the oncoming car (“go”) in 47% and to wait (“stay”) in 53% of left turns. All trials were included in the decision probability analyses, but only “go” trials were included in the response time analyses.

Replicating existing findings from naturalistic studies of driving (Davis & Swenson, 2004; Patil & Pawar, 2014), we found that probability of a decision to go increased with initial time-to-arrival (*z* = 9.1, *p* < 0.001) and initial distance (*z* = 9.4, *p* < 0.001) to the oncoming vehicle (Table 2). The existing literature does not report on timing of gap acceptance; interestingly, we found that response time increased with initial time-to-arrival (*t* = 4.7, *p* < 0.001), but there was no evidence that it was affected by initial distance (*t* = −1.4, *p* = 0.16) (Table 3).

**TABLE 2:** Results of Statistical Analysis of the Effect of Distance and TTA Conditions on Decision. The Binomial Mixed-Effects Model Included Random Slopes of Distance and TTA Conditions per Participant.

	Estimate	Std. Error	z value	Pr( >| z|)
Intercept	−0.28	0.28	−1	0.31
TTA condition	1	0.11	9.1	5.7 × 10^−20^
Distance condition	1.4	0.15	9.4	5.2 × 10^−21^

**TABLE 3:** Results of Statistical Analysis of the Effect of Distance and TTA Conditions on Response Time in “Go” Decisions. The Linear Mixed-Effects Model Included Random Intercept per Participant.

	Estimate	Std. Error	Df	t value	Pr( >| t|)
Intercept	−0.63	0.02	16	−31	1.7 × 10^−15^
TTA condition	0.023	0.0049	8.7 × 10^2^	4.7	3.1 × 10^−6^
Distance condition	−0.0072	0.0052	8.7 × 10^2^	−1.4	0.16

## EVIDENCE ACCUMULATION MODEL OF GAP ACCEPTANCE DECISIONS

**Figure 4.**
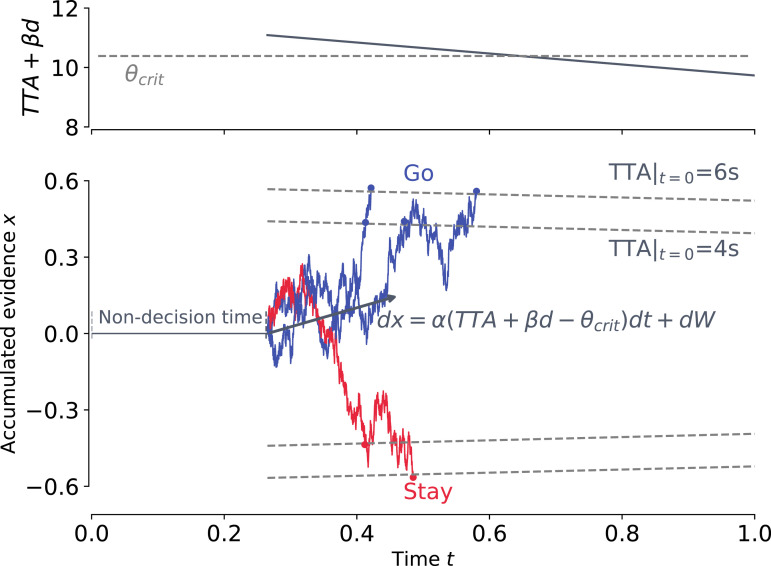
Diagram of the drift-diffusion model of driver’s decision making. Relative evidence in favor of going over staying is accumulated until it reaches either a positive or a negative boundary, at which point a “go” or “stay” decision is made, respectively. The rate of accumulation depends on a linear combination of the current values of TTA and *d* (upper panel). Boundaries for two TTA conditions are plotted for comparison. For each trace, the markers illustrate the moment when the decision is made at the boundaries corresponding to TTA|_*t*=0_ = 4s and 6s. For simplicity, non-decision time is illustrated as a perception delay before the start of evidence accumulation; in our model, non-decision time also accounts for the response delay after the decision is made. Time-varying perceptual evidence determines accumulation rate.

We hypothesized that the decision-making process of the driver when choosing whether to stay or go is based on noisy integration of sensory evidence. This integration is subject to noise, and is terminated when sufficient evidence is accumulated. Such decision-making processes are often described by the drift-diffusion model (Bogacz et al., 2006; Ratcliff & McKoon, 2008), which we adopt as a basis for our model. However, the complexity of our gap acceptance task poses fundamental challenges for the classical drift-diffusion model and the likes (see online supplementary information). We address this issue by generalizing the drift-diffusion model in two ways: first, the evidence accumulation rate is determined by a time-varying perceptual evidence, and, second, the amount of evidence that needs to be accumulated decreases with closing time gap (Figure 4).

First, in our left-turn scenario, the incoming evidence dynamically varies over the time course of each decision. In this scenario, the main perceptual quantities driving the drivers’ decisions are distance and time-to-arrival (Table 2, see also Davis and Swenson (2004)). However, unlike typical laboratory experiments on decision making, these perceptual quantities systematically and substantially change during several hundreds of milliseconds needed to make a decision. Therefore, the canonical assumption of fixed accumulation rate does not hold in our case. To address this, our model hypothesizes that the accumulation rate dynamically depends on distance and time-to-arrival, and is therefore time-dependent.

Assuming that evidence accumulation is driven by a linear combination of distance and time-to-arrival (Giles et al., 2019), its dynamics can be described by the stochastic differential equation

(1) $$dx=\alpha \left(\left(\text{TTA}+\beta d\right)-\theta_{\text{crit}}\right)dt+dW$$

where *x* is the decision variable, TTA = TTA(*t*) is the time-to-arrival of the oncoming car at time *t*, *d* = *d*(*t*) is the distance to the oncoming car at time *t*, and *W* is a stochastic Wiener process. The drift rate parameter *α* ≥ 0 quantifies the relative contribution of incoming perceptual information to the accumulated evidence (decisions are made at random if *α* = 0). Relative weighting of distance information (compared to time-to-arrival) is characterized by parameter *β*. Parameter *θ*_crit_ determines the “critical” value of TTA + *βd*, such that at the time TTA + *βd* = *θ*_crit_, the drift rate changes sign.

### Task Constraints Drive Urgency Effect Via Collapsing Boundaries

Second, with the oncoming car approaching the intersection, the window of opportunity for the driver to go closes fast, which inherently creates an urgency effect: When the time-to-arrival of the oncoming vehicle is large, there is no time pressure for the driver to make a decision, and the driver therefore can sample as much evidence as needed to make an informed decision. However, when the time-to-arrival is small, the driver is compelled to finalize the decision as fast as possible in order to allow enough time for the turning maneuver in case the decision is made to go. Previously, similar time pressure effects in evidence accumulation have been modeled by decision boundaries collapsing over time (Churchland et al., 2008; Drugowitsch et al., 2012). We adopt the same approach in our model, with one important distinction. As the decision urgency in our setup increases with decreasing TTA, we assume the decision boundary to collapse with TTA: the lower the TTA, the less evidence the driver needs to accumulate in order to arrive to a decision.

To capture these considerations, our model assumes that the dynamic accumulation process (1) is terminated when evidence *x* hits one of the boundaries

(2) $$b\left(t\right)=\pm b_{0}f\left(\text{TTA}\left(t\right)\right),$$

where *b*_0_ is the boundary scale parameter, and *f*(⋅) is an increasing function of TTA such that

$$f\left(\text{TTA}\left(t\right)\right)\approx \left\{ \begin{array}{cc} 1 & \text{if TTA}\left(t\right)\gg \tau \\ 1/2 & \text{if TTA}\left(t\right)\approx \tau \\ 0 & \text{if TTA}\left(t\right)\ll \tau \end{array}\right..$$

For simplicity, we chose a sigmoid function satisfying these conditions

(3) $$f\left(\text{TTA}\right)=1/\left(1+e^{-k\left(\text{TTA}-\tau \right)}\right),$$

where the parameter *k* > 0 defines the sensitivity of boundary to time-to-arrival, and *τ* is the time-to-arrival at which the boundary is at its baseline value (±1/2*b*_0_).

### Perception and Action Delays

Our model only captures the decision process itself, and does not represent sensory perception and decision execution, and, consequently, any time delays associated with them. These delays are necessarily present in the experimentally measured response times, and are typically modeled by singling out a non-decision component of the overall response time (i.e., response time is the sum of decision time and non-decision time) (Ratcliff & McKoon, 2008; Ratcliff & Tuerlinckx, 2002). We included a normally distributed non-decision time in the model

(4) $$t^{\text{ND}}\in N\left(\mu_{\text{ND}},\sigma_{\text{ND}}\right).$$

### Model Fitting

In total, our model (1)–(4) has eight free parameters: *α*, *β*, *θ*_crit_, *b*_0_, *k*, *τ*, *μ*_ND_, *σ*_ND_. We implemented the model in pyddm (Shinn et al., 2020b), and fitted it to the data using differential evolution optimization of the weighted least-sum score (Ratcliff & Tuerlinckx, 2002).

To assess the explanatory power of the model, we fitted it to the data obtained from each participant individually. In addition, to characterize the typical behavior over all participants, we fitted the model to the group-averaged probabilities and response time distributions. To quantify the group-averaged response time distributions across participants, we used the vincentizing approach (Ratcliff, 1979; Rouder & Speckman, 2004; Vincent, 1912): based on the individual participants’ data, we calculated per-participant RT quantile functions, which were then averaged across participants. The group-averaged cumulative distribution function was then calculated as an inverse of the group-averaged quantile function. The group means of response times and probability of going were calculated as the average of within-participant mean values.

## MODELING RESULTS

### Evidence Accumulation Model Explains and Predicts the Experimental Results

**Figure 5.**
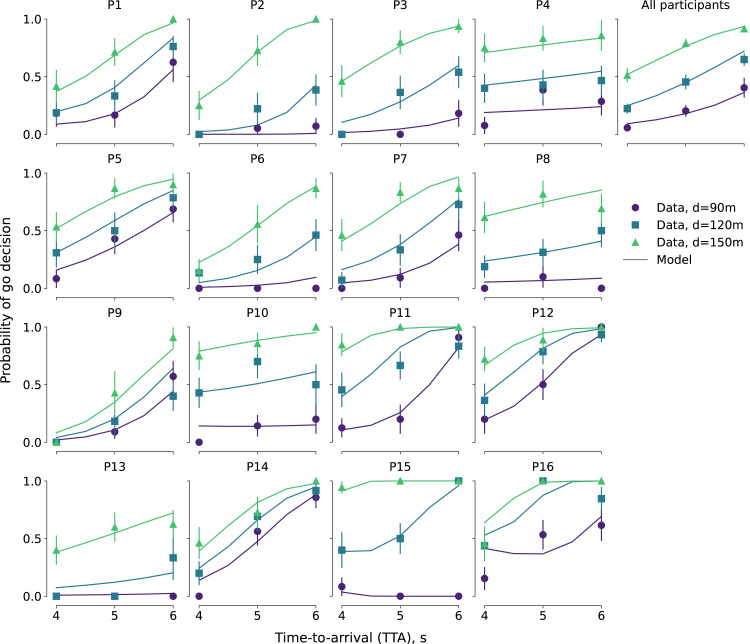
Probability of “go” decision as a function of distance and time-to-arrival of the oncoming car: Individual participants’ and group-averaged data (markers) and behavior of the model fitted to the respective datasets (lines). The markers in the “all participants” panel represent probabilities averaged over within-participant mean values. Error bars denote binomial proportion standard error of mean.

The model explained the observed effect of distance and time-to-arrival on probability of a go decision (Figure 5). Not all participants were described equally well, in particular, the behavior of participant 16 posed a problem for the model. However, overall the model exhibited a full range of behaviors observed in the participants, capturing the behavior of participants whose decisions were relatively weakly affected by the distance condition (e.g., participant 14), as well as those who seemed to decide mostly based on the distance and not time-to-arrival (e.g., participants 10 and 13).

**Figure 6.**
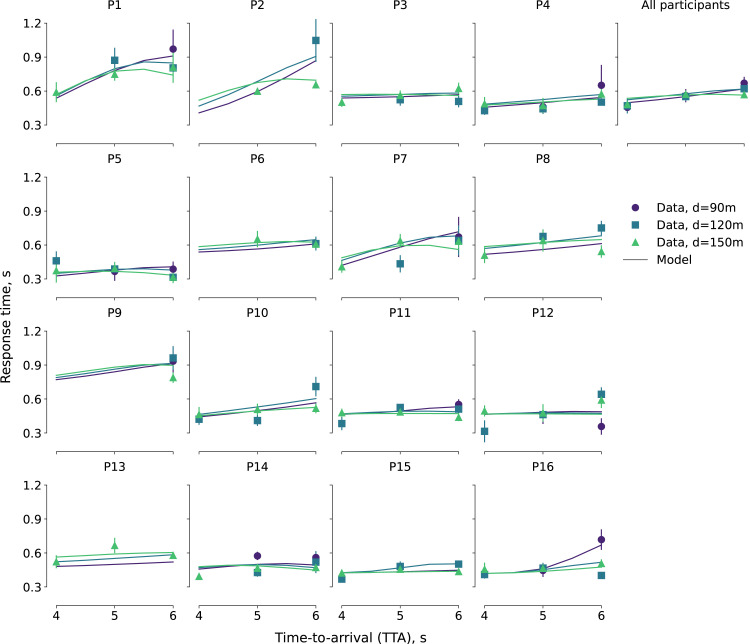
Response time in the “go” decisions as a function of distance and time-to-arrival of the oncoming car: Individual participants’ and group-averaged data (markers) and behavior of the model fitted to the respective datasets (lines). The markers in the “all participants” panel represents response times averaged over within-participant mean values. Response times for conditions with less than four data points are omitted. Error bars indicate standard error of mean.

Crucially, the model captured the observed response times of all sixteen drivers, as well as the group-averaged response times. Specifically, the model explained the positive relationship between response time and time-to-arrival in the group-averaged data (Figure 6). Notably, the model could capture a range of the effect sizes across participants (e.g., participant 1 vs participant 3).

**Figure 7.**
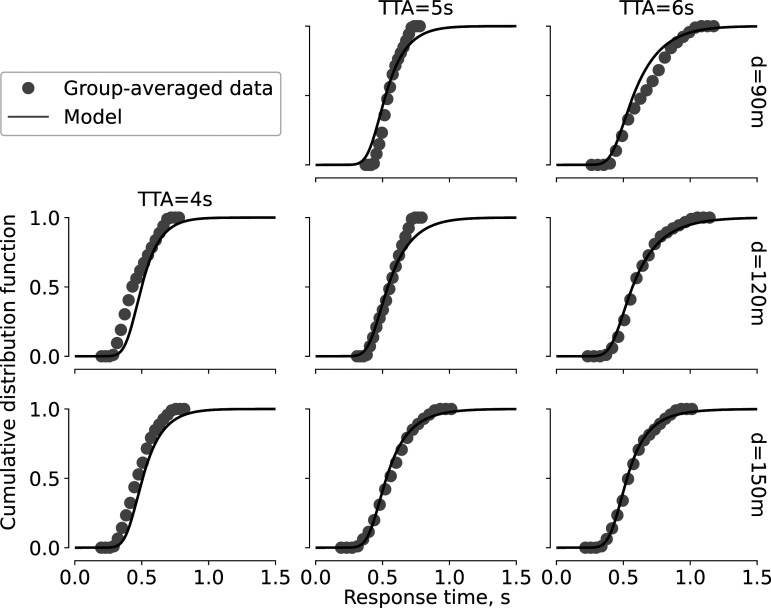
Full distributions of response time in “go” decisions: group-averaged cumulative distribution functions (markers) and distributions generated by the model fitted to the group-averaged response times (solid lines). Distribution for TTA = 4s, *d* = 90m is not visualized because all participants together made only 11 “go” decisions in this condition.

Full response time distributions provide further evidence supporting our model. The model fitted to the group response times generated cumulative distribution functions which followed the group-averaged estimates in eight out of nine conditions (Figure 7). In the remaining condition with the shortest TTA and smallest distance (TTA = 4s, *d* = 90m) however, there were not enough “go” responses to reconstruct the full distribution (only 11 response times across 16 participants). The match between the group-averaged distributions and the model in all other conditions however indicates that the model did not simply fit the “go” probability and mean response times, but also captured the full range of response times generated by the participants.

In all analyses above, the model was fitted to all data available from an individual participant, or the group-averaged data. This allowed us to assess whether the model could explain the observed behavior, but not whether it generalizes beyond the data used for fitting, which would not be the case, for instance, if the model was overfitted.

To investigate how the model generalizes to out-of-sample conditions, and at the same time highlight any potential cases of overfitting, we performed cross-validation of the model using a hold-one-condition-out procedure, focusing on the group-averaged data. For each of the nine (TTA, *d*) conditions, we fitted the model to the data from the remaining eight conditions. The fitted parameters were then used to predict the decision outcomes and mean response times in the ninth, held-out condition. This was repeated nine times, for nine held-out combinations of time and distance gaps.

**Figure 8.**
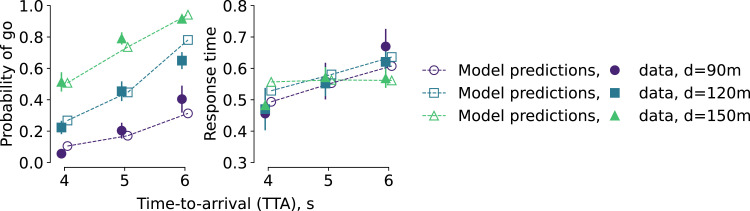
Hold-one-condition-out cross-validation of the model. Filled markers represent the group-averaged data (same as in the “all participants” panels in Figures 5 and 6). Hollow markers indicate model-generated predictions for the held-out condition. Dashed lines connect the hollow markers for visual clarity and do not represent outputs of the model.

The model predictions in this cross-validation setting still matched the observed decision probabilities and response time (Figure 8), albeit less well than when fitting to full data. Of all conditions, the response times predicted by the model deviated furthest from the data for TTA = 4s, *d* = 150m. This indicates a certain degree of overfitting, possibly highlighting that this condition (yielding in a somewhat extreme oncoming car speed of 37.5 m/s) is close to the edge of the model’s feasibility scope. Notably, for other conditions excluding parts of the data from the training set did not substantially affect the simulated model behavior and therefore the match between its predictions and the data. Overall, the cross-validation results suggest that the model’s predictions of the “average” participant’s behavior can generalize to out-of-sample conditions.

### Comparison to Simpler Evidence Accumulation Models

**Figure 9.**
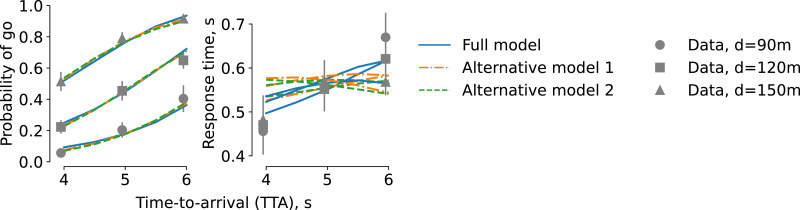
Comparison of the model with simpler alternative models. Alternative model 1: basic drift-diffusion model (constant drift rate, constant decision bound); Alternative model 2: time-varying drift rate, constant decision bound.

To investigate whether our observations could be explained by simpler evidence accumulations models, we have fitted two simplified versions of our model to the data (Figure 9, see also online supplementary information). The first alternative model had the constant drift rate and constant decision bound; the second one included time-varying drift rate but constant decision bound. Both these models could account for the decision outcome data as accurately as the model considered here. However, these models failed to capture the observed effect of time-to-arrival on response time. This suggests that both the time-varying evidence accumulation rate and collapsing decision bounds play an essential role in the participants’ behavior.

## DISCUSSION

Human drivers make high-stakes decisions on a daily basis; understanding and predicting these decisions can facilitate the design of safe and efficient transportation systems. To develop such an understanding of left-turn decisions, we build upon the classical drift-diffusion model, and extend it with two key notions. First, our model has a variable drift rate linked to the relevant time-varying perceptual quantities—distance and time-to-arrival to the oncoming vehicle—which are dynamically sampled from the environment. Second, the model relies on collapsing decision boundaries, reflecting the impending constraints imposed on the decision execution by the environment. The model explained the observed decision outcomes and response times, as well as substantial individual differences in both. Cross-validation demonstrated that the model not only explained the data, but also generalized to out-of-sample conditions, effectively providing a way to predict human drivers’ behavior.

Models of gap acceptance have traditionally focused only on the final outcome of a decision, and the factors affecting it (Davis & Swenson, 2004; Farah et al., 2009; Hamed et al., 1997; Toledo, 2007). Our work adds to this literature by emphasizing the *process* of a decision. In practice, process models of traffic decisions yielding predictions of response times can help to optimize safety and traffic flow efficiency (Markkula, Romano, et al., 2018). More fundamentally, modeling the process of drivers’ decisions provides an insight into the mechanisms of decision making, contributing to theoretical understanding of driver behavior.

Our study is not the first to suggest that evidence accumulation can play a role in behavior on the road; models of this nature have been applied to driver steering and braking decisions during basic sensorimotor control of the vehicle (Markkula, Boer, et al., 2018; Ratcliff, 2015; Ratcliff & Strayer, 2014; Xue et al., 2018), as well as to more complex decisions such as pedestrian road crossing (Giles et al., 2019; Markkula, Romano, et al., 2018). Some of this research has achieved high methodological rigor, on par with the laboratory research on decision making, by collecting large number of data points per participant (Ratcliff, 2015; Ratcliff & Strayer, 2014). To make this possible, these studies used paradigms with speeded response to discrete-onset stimuli, such as brake lights, thus not addressing the types of continuously time-varying sensory information that is crucial in many aspects of driving (Markkula et al., 2016; Toledo, 2007). Other work has considered time-varying sensory information, but has not been as rigorous in the model-fitting approach, for example, absorbing both intra-individual and inter-individual variability into the evidence accumulation noise (Giles et al., 2019; Markkula, Boer, et al., 2018; Xue et al., 2018). The crossing behavior modeling study most similar to ours was that of Giles et al. (2019), who aimed to capture pedestrian crossing decisions. They fitted a number of model candidates to the empirical data, but by own admission they were not able to draw firm conclusions due to limitations in their model-fitting approach. Therefore, to the best of our knowledge, our study is the first to demonstrate methodologically stringent evidence for the value of this type of decision-making model in a driving task with continuously varying sensory evidence.

One of the key notions highlighted by our results is that decisions based on time-varying evidence can be modeled by a drift-diffusion model with the drift rate directly linked to the relevant dynamic perceptual quantities in the environment. This goes beyond the traditional approaches to decision making. These approaches almost ubiquitously focus on the stimuli with the magnitude which is constant throughout the trial, which is naturally modeled by evidence accumulation with constant drift rate (Bogacz et al., 2006; Ratcliff & McKoon, 2008; Ratcliff et al., 2016). In laboratory setting, variable-drift evidence accumulation is rarely investigated, mostly as a way of modeling urgency signals (Cisek et al., 2009; Murphy et al., 2016), non-uniform temporal weighting of evidence throughout the trial (Bronfman et al., 2016; Eckhoff et al., 2008), and artificially introduced perturbances in sensory evidence (Cheadle et al., 2014; Shinn, Ehrlich, et al., 2020). The previous studies on action initiation in intermittent motor control (Markkula, Boer, et al., 2018; Zgonnikov & Markkula, 2018) and the early evidence accumulation based models of traffic interactions discussed above (Giles et al., 2019; Markkula, Romano, et al., 2018) highlighted the promise of the variable-drift approach. However, due to complexity of the analyzed tasks these studies could not disentangle the effects of evidence accumulation and other mechanisms at play. Reinforcing these initial results, our study provides conclusive evidence that evidence accumulation with variable drift can explain gap acceptance decisions made based on naturalistic time-varying stimuli.

Our results also emphasize the crucial role of time pressure induced by the impending oncoming car. The experimental data revealed that response times increased with time-to-arrival in most participants (Figure 6). This goes against predictions of drift-diffusion models with fixed boundaries that imply faster response times for a given decision (in our case, “go”) when perceptual information from the environment (in our case, long TTA) favors that decision. The mechanism of our model which allowed it to capture this experimental observation is decision boundaries collapsing with time-to-arrival. Our scenario is therefore interestingly different from typical speed-accuracy trade-off paradigms in decision-making research, in which time pressures are external to the task being performed (Churchland et al., 2008; Drugowitsch et al., 2012; Shinn, Ehrlich, et al., 2020). In contrast, in the left-turn decisions, the perceptual information being decided upon (time-to-arrival) simultaneously dictates the urgency. Indeed, the time gap which is closing fast imposes an inherent dynamic constraint on decision execution, which the driver has to take into account when deciding.

Our work goes beyond the decision outcomes and begins to uncover the evidence accumulation mechanisms behind the left-turn decisions, but we consider it to be just the first step towards a comprehensive account of drivers’ decision making in gap acceptance. Future work should address the key limitation of our study—the lack of a reliable proxy for the duration of the decision process in “stay” decisions. Traditionally, response times for both options are used to constrain the fits of evidence accumulation models. However, in our experiment, the measurement of “stay” response times could not be extracted from the vehicle motion data. This could potentially be aided, for example, by asking the participants to press a button as soon as they decide to stay. We however opted for not doing so, because such a button press would not be as automatized a behavior as pushing the gas pedal, which could lead to inflated non-decision time in the “stay” decisions and interfere with the decision-making process in “go” decisions. Follow-up studies should develop ecologically valid ways of capturing “stay” response times.

Duration of a decision process provides a useful but coarse-grained measurement of a cognitive process. Going beyond response times, dynamic measures such as movement trajectories can provide researchers with rich data about a cognitive process (Schulte-Mecklenbeck et al., 2017; Zgonnikov et al., 2019). This includes, for instance, information on gradual or abrupt reversal of preference prior to commitment to the final decision (changes-of-mind). Our data indicates that up to 15% of “go” trials could potentially include a change-of-mind when a driver first accelerates soon after seeing the oncoming car, but then stops short of taking the turn, waiting until the car passes instead. However, within our experimental procedure, such changes-of-mind cannot be reliably distinguished from “rolling” behaviors when the drivers simply inch forward after making the “stay” decision in order to take the turn faster after the oncoming vehicle had passed. This is further complicated in case of changes from “stay” to “go,” which cannot be identified based on vehicle motion data alone. Developing a way of reliably identifying changes-of-mind can enable modeling them, which can in turn clarify to what extent they are caused by continued accumulation of new evidence (Fleming et al., 2018; Resulaj et al., 2009) or high level of uncertainty about the initial decision (Atiya et al., 2019; Atiya et al., 2020).

A general limitation of the evidence accumulation models which also extends to our model is the lack of mechanistic explanations of post-decision processes such as decision execution (Evans & Wagenmakers, 2019). In the context of our task, this means that our model cannot account, for example, for the rate of acceleration applied after the “go” decision is made. One possible approach to extend our model in this direction could be through decision confidence (Kepecs et al., 2008; Kiani & Shadlen, 2009): hypothetically, the more confident the driver is in their “go” decision, the stronger they press the gas pedal to execute it. However, this approach requires further experimental investigations to collect per-trial confidence judgments in a non-intrusive manner. Furthermore, it is not clear whether the drift-diffusion model is an adequate framework for capturing decision confidence (Ratcliff et al., 2016; Yeung & Summerfield, 2012). Future studies should investigate the relation between evidence accumulation and response execution in drivers’ decisions both experimentally and computationally.

In addition to theoretical insight, our work has potential practical applications for implementing human models in real-time prediction of human behavior in traffic, as well as virtual environments for testing automated vehicles. Most research on real-time human prediction in traffic uses computationally convenient but cognitively implausible models of human behavior, for instance, fixed decision probability (Kooij et al., 2019; Thornton et al., 2019) and utility maximization (Sadigh et al., 2018; Schwarting et al., 2019), which is known to diverge substantially from actual human behavior (Kahneman & Tversky, 1979), including that in driving (Schmidt-Daffy, 2014; Siebinga et al., 2022). Therefore, having cognitively grounded models of human behavior in traffic interactions can help to generate more accurate and generalizable predictions of road user behavior. Our study provides a step forward here, but needs to be complemented with further work on other scenarios, and translated to real-world contexts with higher variability than in typical controlled studies.

Taken together, our results suggest that dynamic evidence accumulation underlies left-turn gap acceptance decisions in human drivers. More generally, this study exemplifies how simple cognitive process models can help us to understand human behavior in complex real-world tasks.

## SUPPLEMENTAL MATERIAL

Supplemental Material - Should I Stay or Should I Go? Cognitive Modeling of Left-Turn Gap Acceptance Decisions in Human Drivers Supplemental Material for Should I Stay or Should I Go? Cognitive Modeling of Left-Turn Gap Acceptance Decisions in Human Drivers by Arkady Zgonnikov, David Abbink, and Gustav Markkula in Human Factors
