# Supplementary material for: Should I Stay or Should I Go? Cognitive Modeling of Left-Turn Gap Acceptance Decisions in Human Drivers
Source: Hum Factors. 2022 Dec 19;66(5):1399–413. doi: 10.1177/00187208221144561 (PMC10958748; doi:10.1177/00187208221144561)
Supplement: Supplemental Material - Should I Stay or Should I Go? Cognitive Modeling of Left-Turn Gap Acceptance Decisions in Human Drivers [file sj-pdf-1-hfs-10.1177_00187208221144561.pdf]

Supplementary information for "Should I stay or should I go? Cognitive modeling of left-turn gap acceptance decisions in human drivers"

Arkady Zgonnikov[1,2], David Abbink[1], and Gustav Markkula[3]
[1]Department of Cognitive Robotics, Faculty of Mechanical, Maritime and Materials Engineering, Delft University of Technology, Netherlands
[2]AiTech, Delft University of Technology, Netherlands
[3]Institute for Transport Studies, University of Leeds, UK

Fitted model parameters

Figure 1 and Table 1 represent the parameters of the models fitted to individual participants as well as the group-averaged data ("all" in Table 1 and square markers in Figure 1) for reference.

To investigate reliability of model parameter estimates, we have checked if the values of the parameters change when we repeat the fitting procedure 10 times (Fig. 2). Because fitting takes a lot of time (around 2-3 hours per fit on an i7 CPU), we do this only for two participants. We chose the participants with the highest and the lowest value of the loss function (P15 and P16, respectively).

Analyses of alternative evidence accumulation models

To investigate if the experimental observations could be explained by simpler evidence accumulation models, we fitted two alternative models to the same data and compared it to the model presented in the main paper. Thus, the three models analyzed are:

- Model 1 (the model presented in the main paper): dynamic drift depending on distance and TTA and dynamic decision boundary which collapses with TTA;
- Model 2 (analogous to the generalized drift-diffusion model proposed by Giles et al 2019): dynamic drift depending on distance and TTA to the oncoming car and constant decision boundary;
- Model 3 (analogous to the standard drift-diffusion model): static drift determined by the values of distance and TTA at the decision onset and constant decision boundary.

We found that the behavior of models 2 and 3 in terms of probability of "go" decision closely followed model 1 (Figure 3). With the exception of participant 16, both simpler models explained the data as well as model 1, suggesting that response times are critical in distinguishing between competing cognitive process models.

In particular, our key experimental observation, the fact that response times increased with TTA, proved difficult to capture for models that do not include a collapsing boundary (Figures 4). The best fits of models 2 and 3 could at best produce RTs that did not change with TTA; the latter was the case for a number of participants (e.g. P3, P6) but the majority of them demonstrated a positive relationship between TTA and RT, which is also captured in the group-averaged data - the latter could only be explained by Model 1. The full RT distribution analysis confirmed that model 1 described the data better than the simpler models (Fig. 5).

Finally, we cross-validated three models using the leave-one-condition-out approach described in the main paper. The results suggest that the two models without the collapsing boundary have a tendency to overestimate response times in the smallest TTA condition and underestimate it in the largest TTA condition, resulting in RT being a non-increasing function of TTA which is counter to the observations (Figure 6).

Analyses of models' predictive validity metrics

To augment the analyses reported in the main paper and investigate the predictive power of the model (as well as two simpler models introduced in the previous section), we evaluated it using the metrics analogous to the ones commonly used for machine learning models. Specifically, area under the receiver operating characteristic curve (AUC) is often used to evaluate predictions of binary variables (in our case, decision outcome). Furthermore, metrics analogous to R^2 from linear regression are useful to compare model predictions of continuous variables (in our case, response time) with the corresponding data.

We note however that these metrics are not straightforward to apply to our case which involves a) nested data - individual measurements are repeated within conditions and within participants, b) the model that is fitted individually to each participant, and c) the model that predicts the full distribution of response times and not only the mean of the observations. We have not found any similar analyses in existing literature on evidence accumulation models, and to the best of our knowledge, there is no generally accepted way of calculating (pseudo) R^2 or AUC under ROC for our kind of data. For this reason, we recommend to interpret the results of this section with caution, and to investigate the validity of these metrics in details in future studies.

Receiver operating characteristic (ROC) curves for model predictions of decision outcome

First, to investigate the theoretical limits of predicting the decision outcomes based solely on TTA and distance conditions, we analyzed ROC curves of the ideal predictor that for each participant "knows" that participant's exact probabilities of “go” decision for each condition (Figure 7). The best-case AUC under ROC curves ranged from 0.75 (P4) to 0.97 (P15) across participants. This indicates that for most drivers, the stochasticity of their decisions places a limit on the accuracy of predicting the decision outcome.

Interestingly, all three models get close to the theoretical limit in terms of the trade-off between the true positive rate and false positive rate (Figure 8), with few exceptions (e.g. P2 for model 2, P16 for all models). This conclusion is reinforced by the ROC analysis

of cross-validated models (Figure 9): the models' ROC curves closely follow the theoretical limit.

These analyses resonate with the results presented in Figure 4, suggesting that even the simplest evidence accumulation models can accurately explain and predict decision outcomes in our scenario.

Pseudo-R_2 of model predictions of mean response time per condition

In order to quantify the correspondence between the model's predicted response times and the experimentally observed ones, we visualized the means of model-predicted response times as a function of the corresponding response times observed in the data (figures 10–12). We did this per condition for each participant, resulting in at most 9 pairs of mean response times per participant (the conditions in which a participant made less than 2 "go" decisions were excluded). We then quantified the relationship by

$$\text{pseudo-}R^2 = 1 - \frac{\sum_{d,\mathrm{TTA}} \left(\overline{\mathrm{RT}}_{\mathrm{model}}(d, \mathrm{TTA}) - \overline{\mathrm{RT}}_{\mathrm{data}}\right)^2}{\sum_{d,\mathrm{TTA}} \left(\overline{\mathrm{RT}}_{\mathrm{data}}(d, \mathrm{TTA}) - \overline{\mathrm{RT}}_{\mathrm{data}}\right)^2}, \quad (1)$$

where $\overline{\mathrm{RT}}_{\mathrm{data}}$ is the mean RT of a participant, and $\overline{\mathrm{RT}}_{\mathrm{model}}(d, \mathrm{TTA})$ is the mean RT of a participant in the condition (d, TTA). This metric the model error relative to the variance of mean RTs across all conditions. In the best-case scenario, the model's predicted mean RTs would be equal to the experimentally observed ones, corresponding to pseudo-$R^2 = 1$, however, when model predictions diverge from the data, pseudo-R^2 can take negative values. The metric (1) resembles the standard R^2 metric used in linear regression models, but cannot be interpreted in the same way (e.g. "fraction of variance explained") due to the inherently more complex modeling approach and hierarchical structure of the data.

Overall, for most participants model 1 showed the highest agreement with the observed response times, compared to models 2 and 3 (Fig. 13). We have analyzed a version of pseudo-R^2 calculated for cross-validated model predictions of group-averaged data (Fig. 14). Reinforcing our conclusions, model 1 was the only model that was able to achieve a positive correlation with the observed mean RTs over all conditions (cf. Fig. 6).

Note that this analysis only provides a limited assessment of the model's predictive power with respect to response times, since it only focuses on mean RTs and ignores potential (mis)matches between the model-predicted and experimental full RT distributions. Future studies can benefit from a metric that captures differences between full RT distributions across all conditions (possibly based on Kullback-Leibler divergence).

Table 1

Model parameters fitted to individual participants and group-averaged data ("all").

Participant	α	β	θ_{crit}	b_0	k	τ	μ_{ND}	σ_{ND}
1	1.47	0.02	6.68	1.36	1.36	3.86	0.26	0.05
2	2.17	0.04	9.47	4.21	0.72	5.94	0.24	0.01
3	2.47	0.05	12.04	0.53	0.11	5.00	0.50	0.14
4	0.21	0.19	26.24	1.15	0.16	4.45	0.22	0.02
5	1.13	0.03	7.57	1.05	0.10	3.98	0.13	0.07
6	2.02	0.04	10.75	0.85	0.20	5.33	0.48	0.11
7	1.20	0.03	7.96	1.33	0.80	3.34	0.12	0.05
8	0.41	0.14	21.63	1.30	0.18	5.14	0.33	0.18
9	1.79	0.01	6.98	1.09	0.11	3.04	0.62	0.16
10	0.45	0.15	22.28	1.05	0.40	5.21	0.32	0.10
11	4.86	0.04	8.85	0.57	0.68	5.54	0.44	0.13
12	4.95	0.02	7.02	0.51	0.46	5.98	0.44	0.18
13	0.69	0.12	21.22	0.95	0.10	3.91	0.38	0.03
14	2.34	0.01	6.11	0.73	0.15	3.18	0.34	0.08
15	4.98	0.16	23.62	0.53	1.98	5.10	0.42	0.10
16	3.22	0.06	10.71	1.53	1.97	5.90	0.41	0.15
all	1.14	0.04	9.89	0.90	0.29	4.15	0.37	0.06

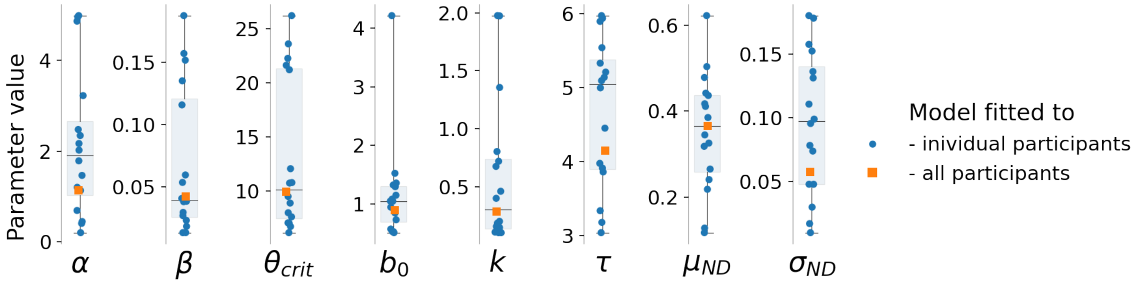

Figure 1

Parameters of the model fitted to the individual participants' data and the group-averaged data ("all participants"). Whiskers denote full ranges of values. Parameter values correspond to those in Table 1

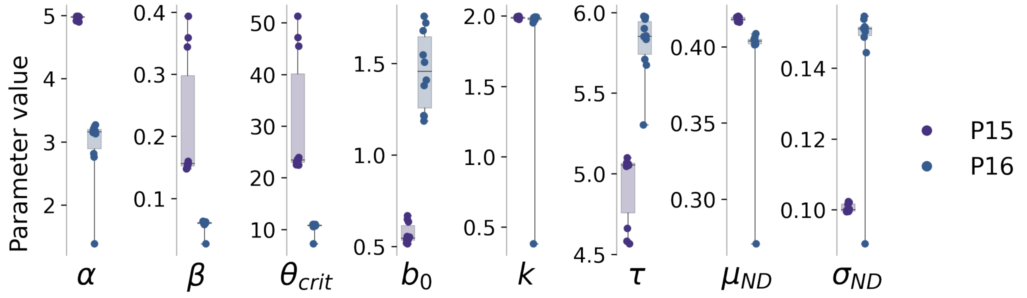

Figure 2

Parameters of the model repeatedly fitted (ten times) to participants 15 and 16. Every data point corresponds to an independent model fit to full data of each participant. Whiskers denote full ranges of values. Median values

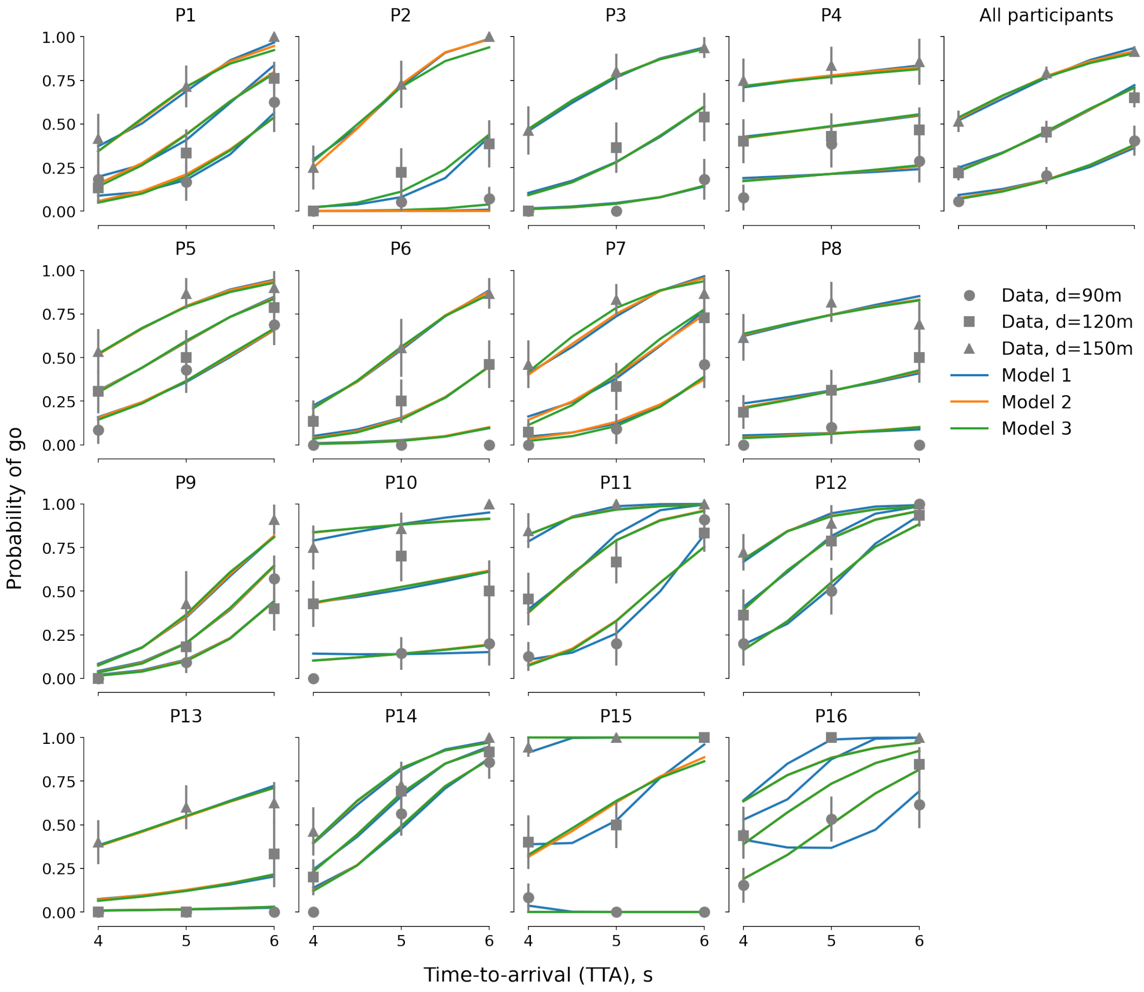

Figure 3

Comparison of model-generated probabilities of "go" decision.

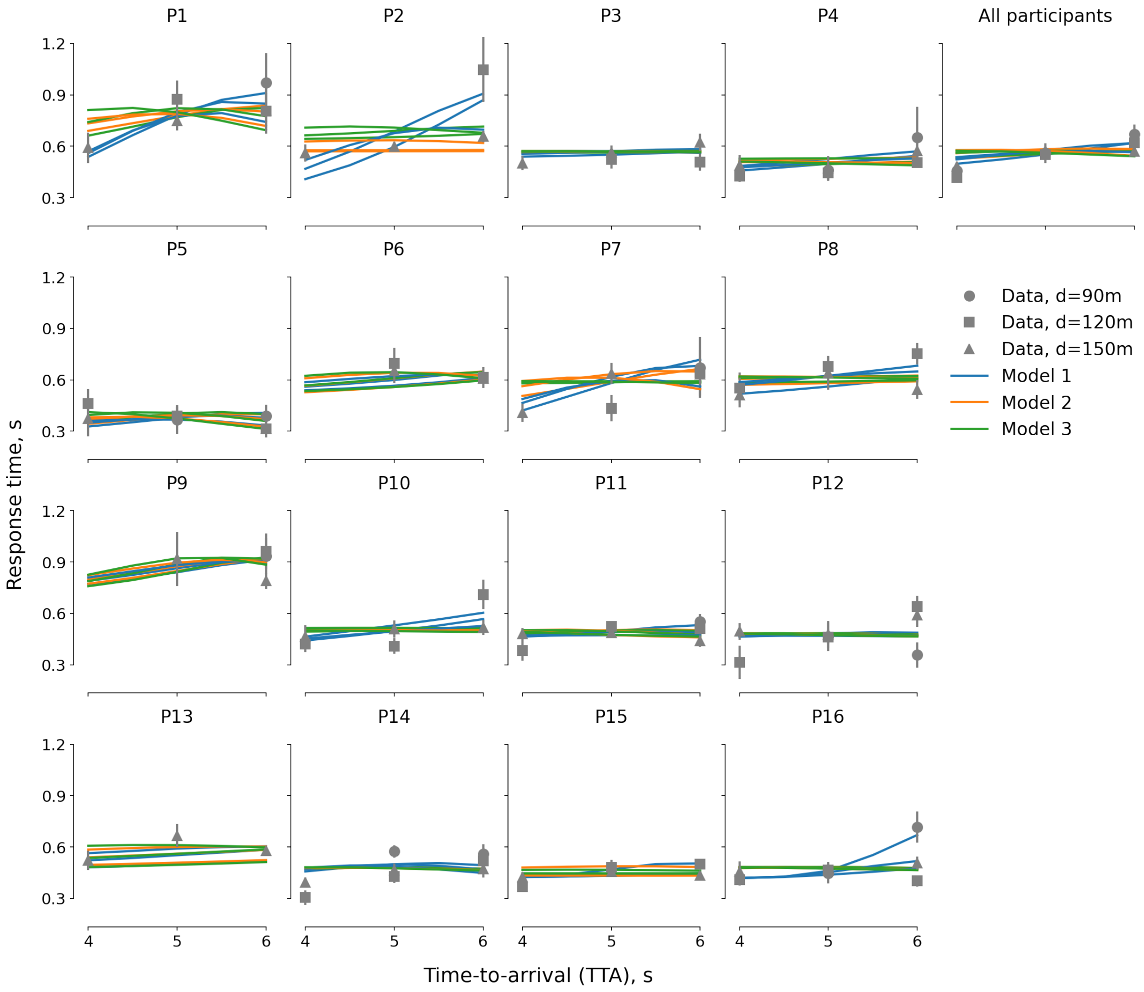

Figure 4

Comparison of model-generated mean response times in "go" decisions.

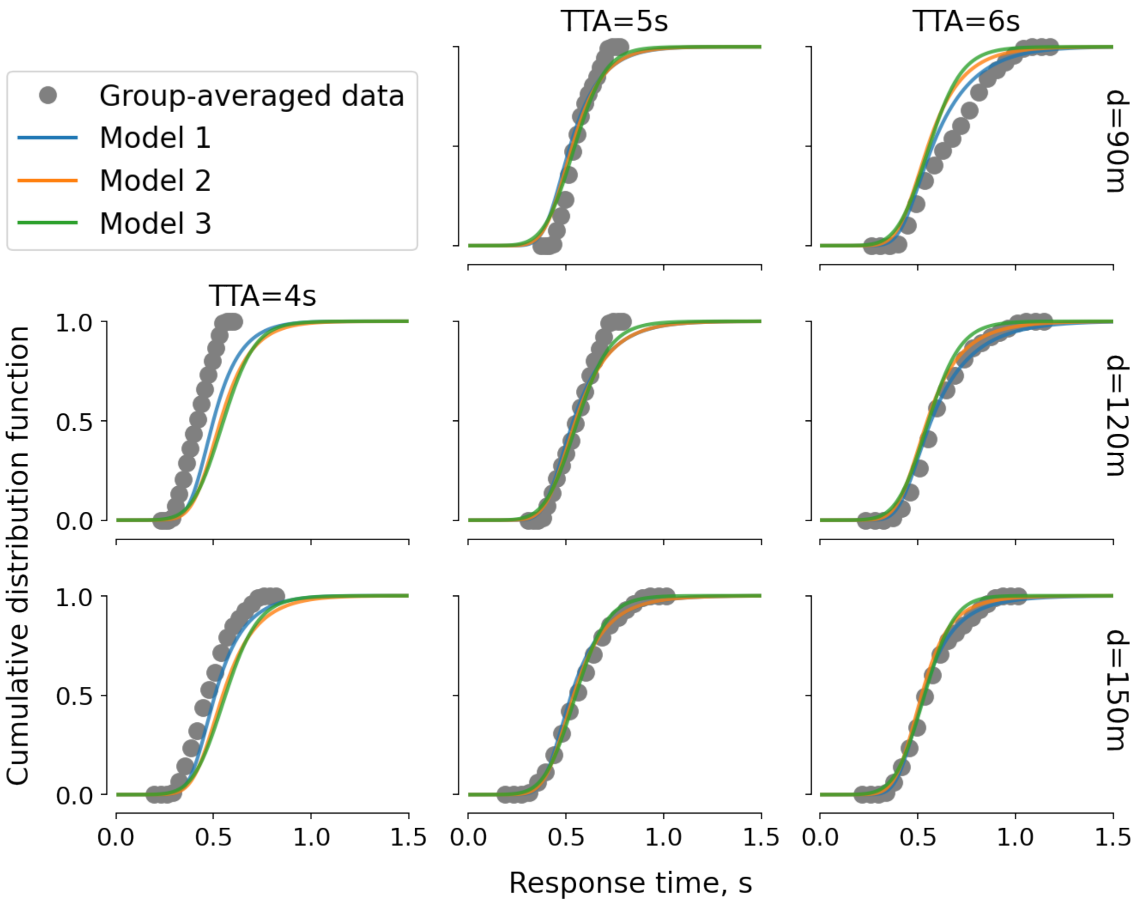

Figure 5

Comparison of model-generated full response time distributions.

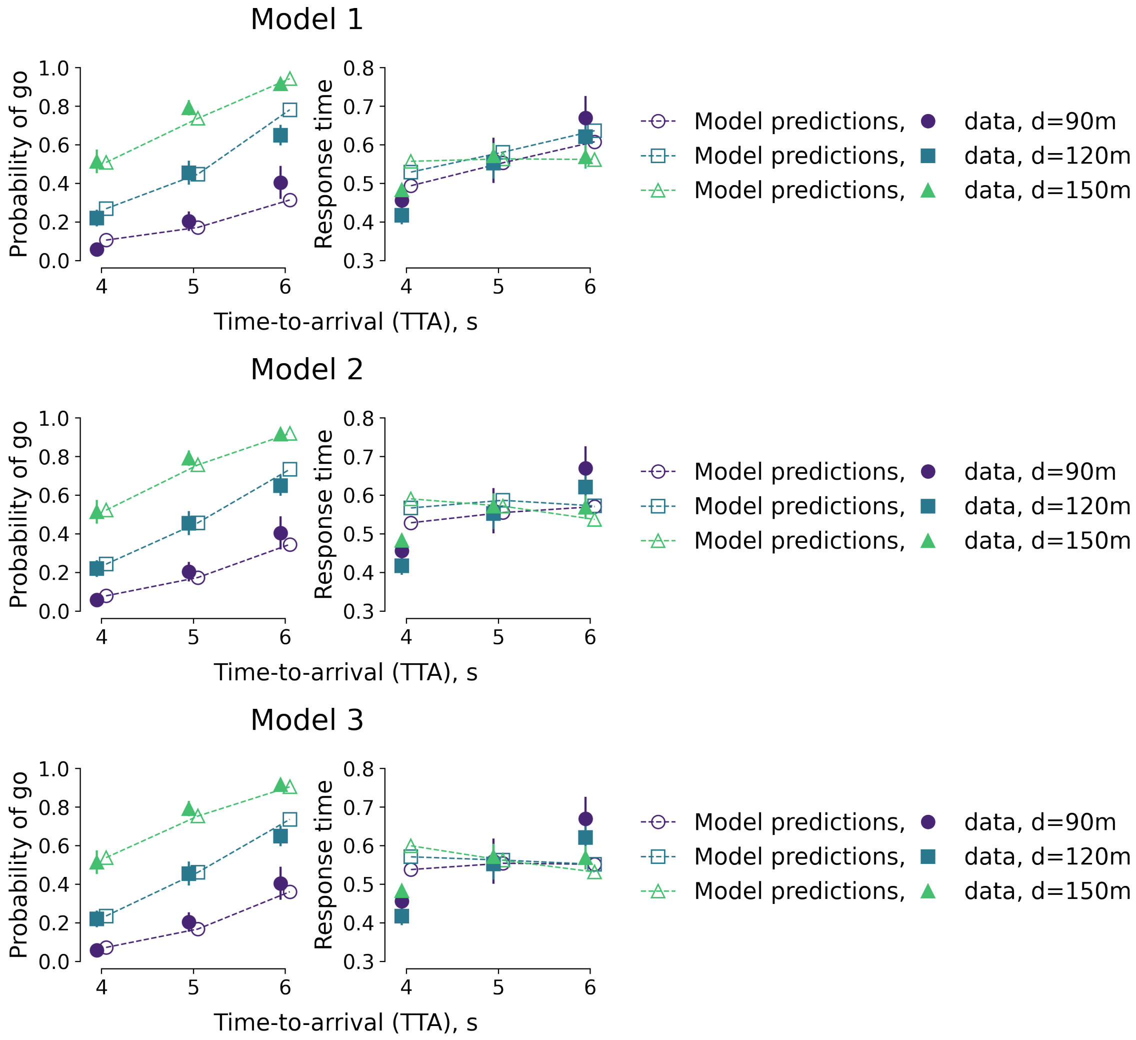

Figure 6

Cross-validation of the three alternative evidence accumulation models.

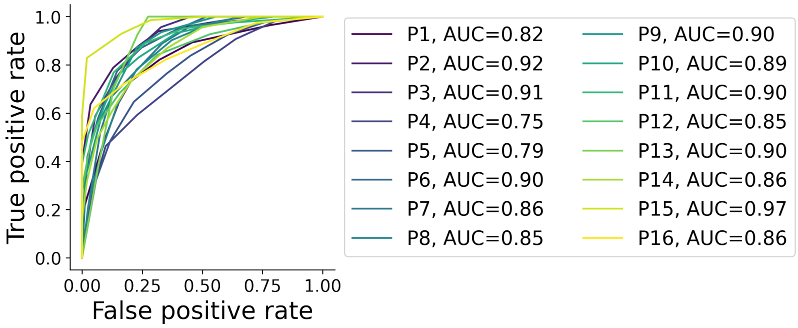

Figure 7

Receiver operating characteristic (ROC) curves and corresponding areas under curve (AUC) for the best-case per-participant predictors that predict the decision outcome based on the experimentally measured probabilities of "go" decision for each condition.

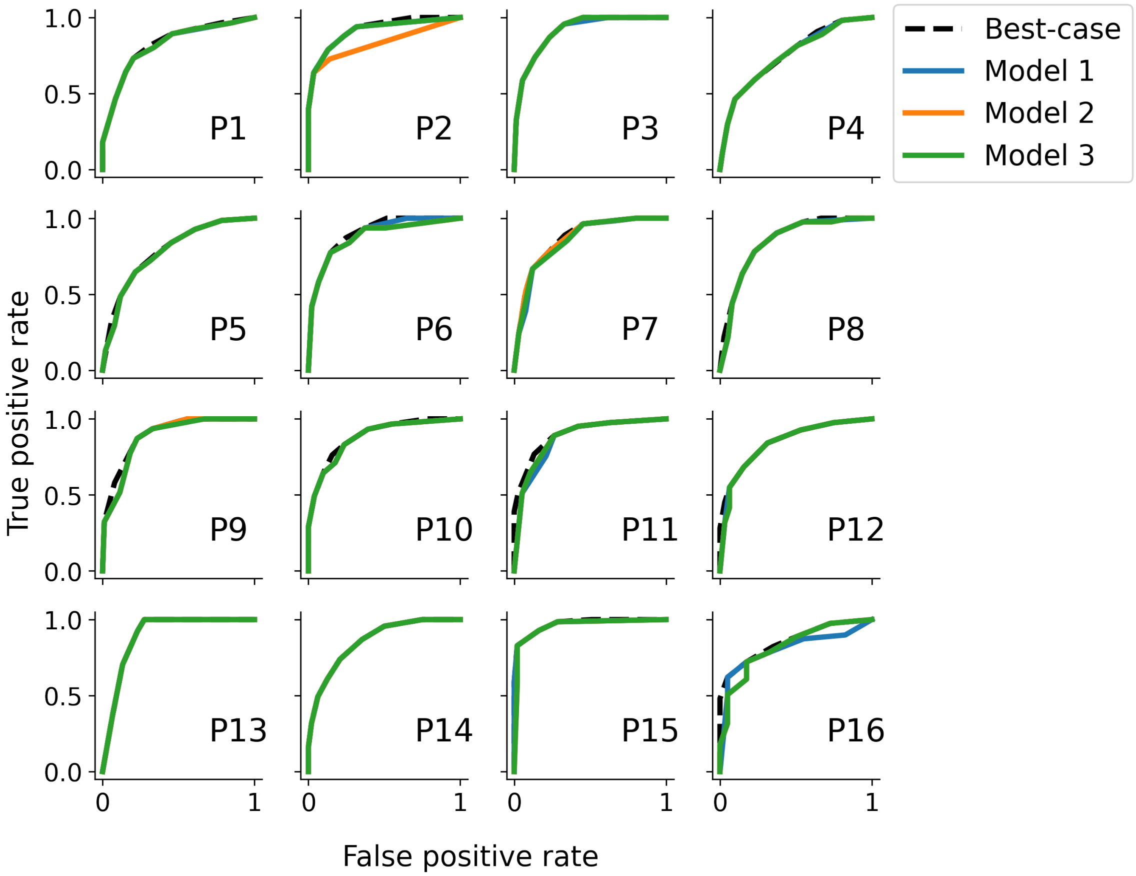

Figure 8

ROC curves and corresponding AUC measures for the three models fitted to individual participants' data, as compared to the best-case predictor for each participant.

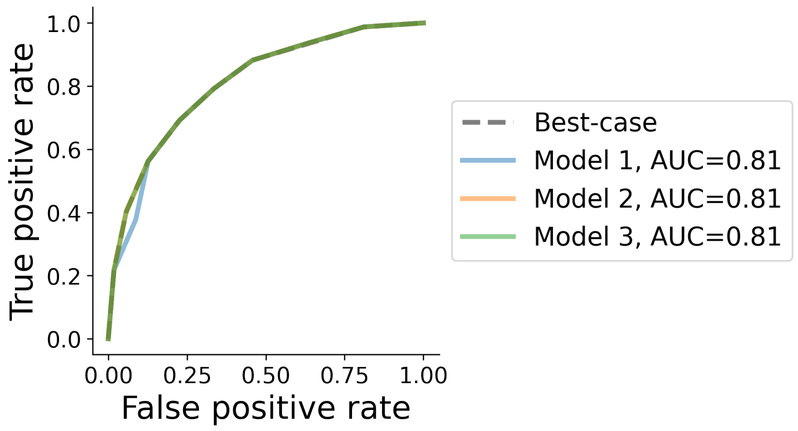

Figure 9

ROC curves and corresponding AUC measures for the three models fitted to the group-averaged data following the cross-validation procedure described in the main paper.

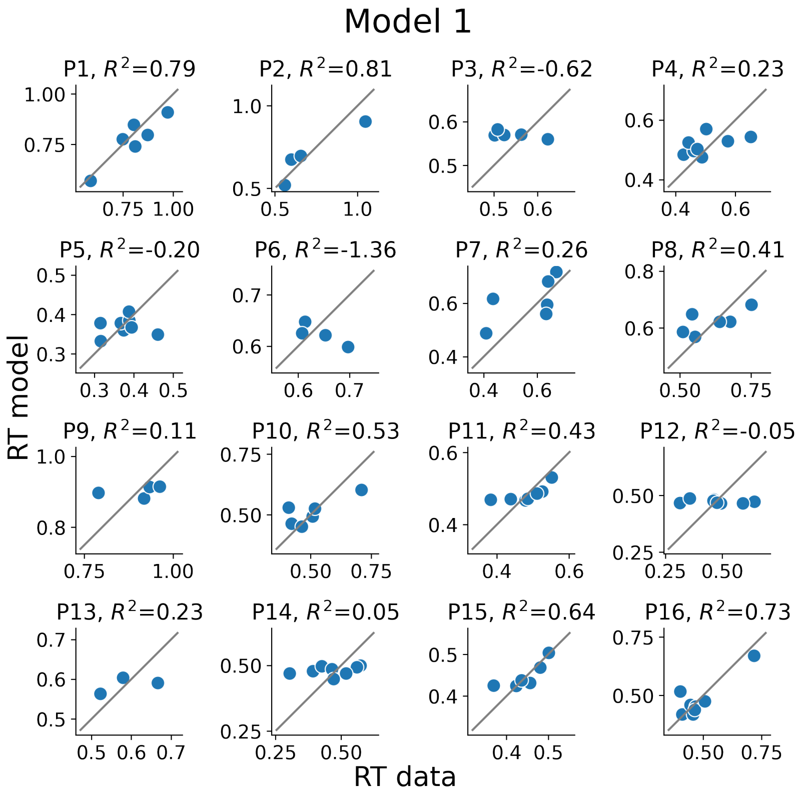

Figure 10

Pseudo-R^2 of model 1 predictions of individual participants' mean response times per condition.

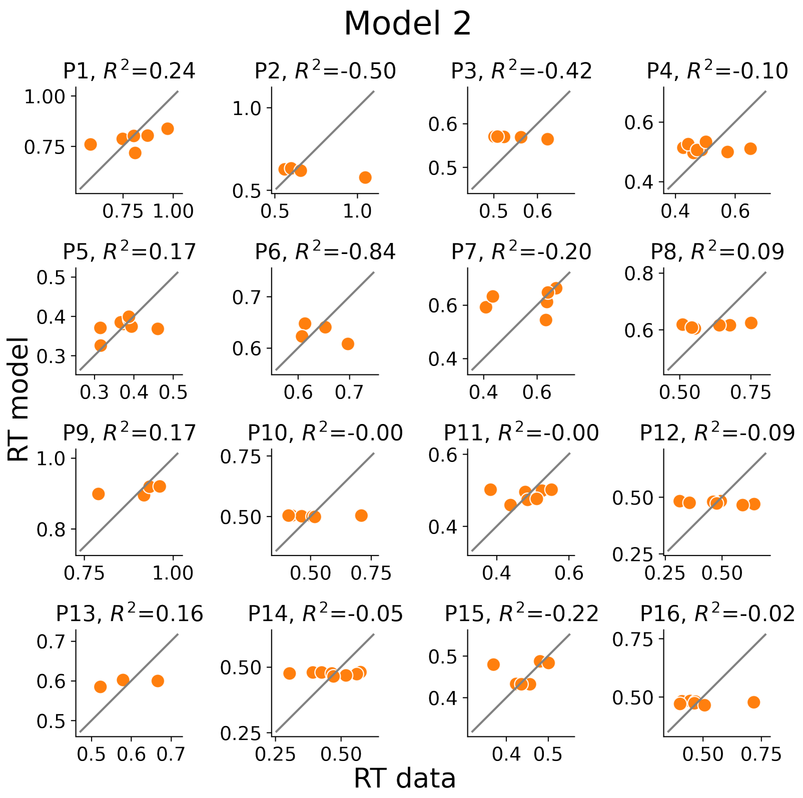

Figure 11

Pseudo-R^2 of model 2 predictions of individual participants' mean response times per condition.

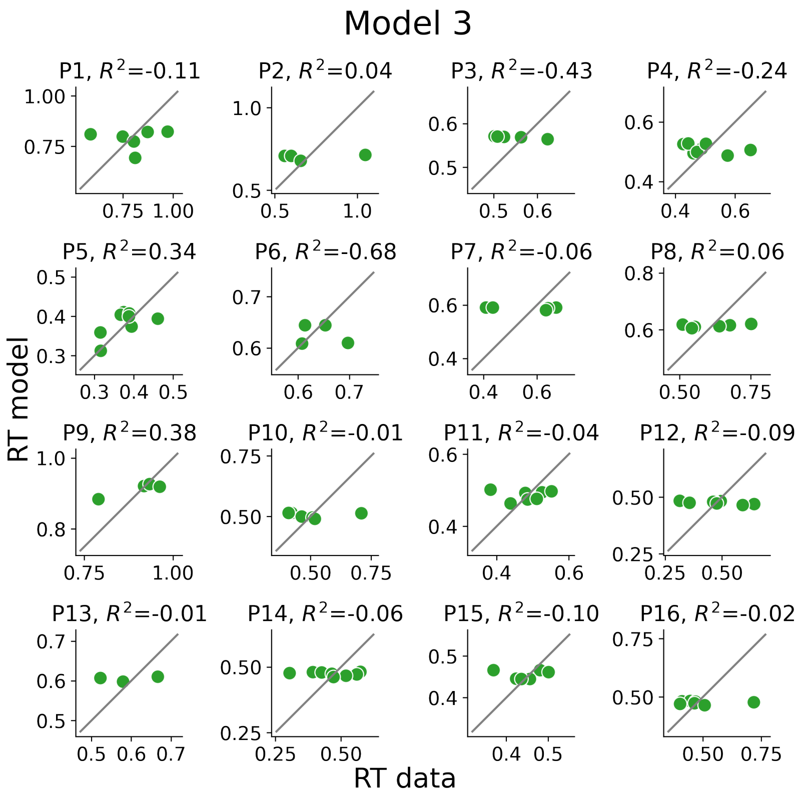

Figure 12

Pseudo-R^2 of model 3 predictions of individual participants' mean response times per condition.

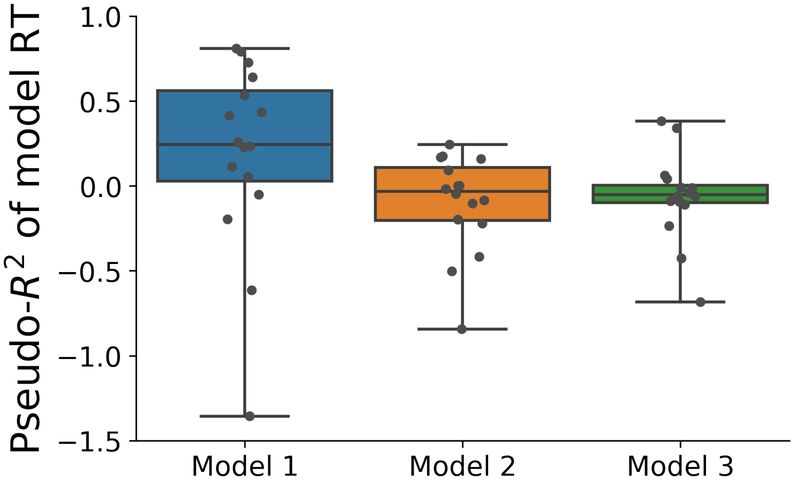

Figure 13

Pseudo-R^2 of model predictions of individual mean response times per condition. Every data point corresponds to one model fit to a single participant's data, i.e., one panel in figures 10–12. Whiskers denote full ranges of values.

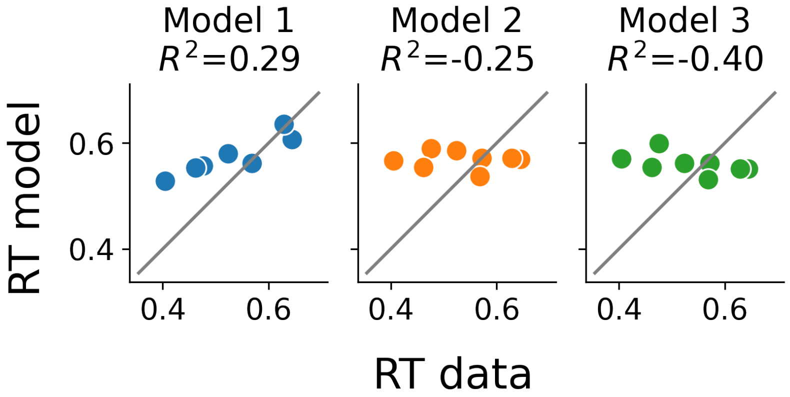

Figure 14

Pseudo-R^2 of cross-validated model predictions of group-averaged mean response times. Every data point corresponds to a model fitted to a combination of 8 conditions.
